# Supplementary material for: Impact of Aβ40 and Aβ42 Fibrils on the Transcriptome of Primary Astrocytes and Microglia
Source: Biomedicines. 2022 Nov 19;10(11):2982. doi: 10.3390/biomedicines10112982 (PMC9688026; doi:10.3390/biomedicines10112982)
Supplement: Supplementary file 1 [file biomedicines-10-02982-s001.zip › biomedicines-1972129-supplementary.pdf]

Supplemental Information

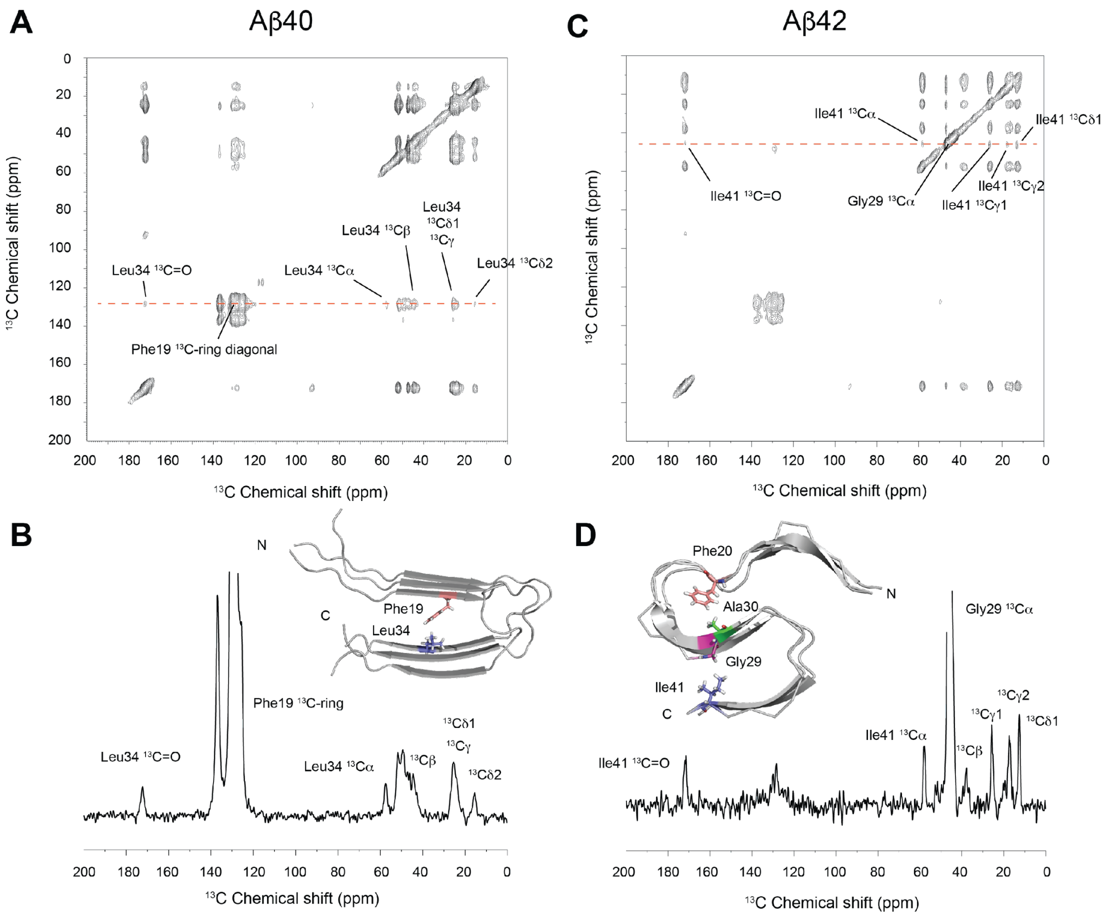

Supplemental Figure S1. 2D ^{13}C solid-state NMR spectra of mature Aβ40 and Aβ42 fibrils. Solid-state magic angle spinning NMR measurements were undertaken on the Aβ40 and Aβ42 fibrils to identify inter-residue contacts that are characteristic of Aβ40 and Aβ42 folds. Measurements of ^{13}C-^{13}C dipolar couplings, which are sensitive to ^{13}C-^{13}C distances between selectively ^{13}C-labeled amino acids, were made using dipolar assisted rotational resonance (DARR). (**A**) 2D DARR NMR spectrum of Aβ40 fibrils incorporating ring-^{13}C-Phe19 and U-^{13}C-Leu34. (**B**) Row through the diagonal resonance of ring-^{13}C-Phe19 at the position of the red dashed line in (**A**) reveal cross peaks with U-^{13}C-Leu34. The labeled Leu34 ^{13}C resonances result from close packing of Phe19 and Leu34 side chains. (**C**) 2D DARR NMR spectrum of Aβ42 fibrils incorporating 2-^{13}C-Gly29 and U-^{13}C-

Ile41. (**D**) Row through the diagonal resonance of 2-^{13}C-Gly29 at the position of the red dashed line in (**C**) reveal cross peaks with U-^{13}C-Ile41. The ^{13}C cross peaks labeled Ile41 result from close packing of Gly29 and Ile41. DARR NMR measurements were also obtained using ring-^{13}C-Phe20 and 3-^{13}C-Ala30 labeled Aβ42 (data not shown). Together with the inter-residue cross peaks in panel (D), the observed Phe20-Ala30 cross peaks are consistent with the "S" shaped monomer within the fibril structure of Aβ42.

The insets of molecular structures in panels (**B**) and (**D**) show 3 or 2 monomers, respectively, within published solution-derived fibril structures of Aβ40 (PDB ID = 2LMP, (Paravastua, Leapman et al. 2008)) and Aβ42 (PDB ID = 2MXU, (Xiao, Ma et al. 2015)). To highlight the close packing interactions between the ^{13}C-labeled residues, only a single protofibril of the full fibril structure is shown. The Aβ40 fibril structures derived from solution are often polymorphic. Paravastua et al. (2008) describe both twisted ribbon and striated ribbon Aβ40 fibril morphologies that exhibit ring-^{13}C-Phe19 and U-^{13}C-Leu34 contacts. The templated growth conditions used in the current study result in twisted ribbons with a cross-over distance of 120-150 nm and width of ~10 nm, consistent the Aβ40 fibrils described by Paravastua et al. (2008) having three-fold symmetry. The three-fold symmetry is similar to that observed in fibrils derived from human brain (Lu, Qiang et al. 2013). The three-stranded Aβ42 structure shown in the inset in panel (**D**) is characteristic of fibrils formed in solution (Colvin, Silvers et al. 2015, Xiao, Ma et al. 2015) and isolated from human brain (Yang, Arseni et al. 2022).

References

Colvin, M. T., R. Silvers, B. Frohm, Y. C. Su, S. Linse and R. G. Griffin (2015). "High resolution structural characterization of Aβ(42) amyloid fibrils by magic angle spinning NMR." Journal of the American Chemical Society **137**(23): 7509-7518.

Lu, J.-X., W. Qiang, W.-M. Yau, C. D. Schwieters, S. C. Meredith and R. Tycko (2013). "Molecular structure of β-amyloid fibrils in Alzheimer's disease brain tissue." Cell **154**(6): 1257-1268.

Paravastua, A. K., R. D. Leapman, W. M. Yau and R. Tycko (2008). "Molecular structural basis for polymorphism in Alzheimer's β-amyloid fibrils." Proceedings of the National Academy of Sciences of the United States of America **105**(47): 18349-18354.

Xiao, Y., B. Ma, D. McElheny, S. Parthasarathy, F. Long, M. Hoshi, R. Nussinov and Y. Ishii (2015). "Aβ(1-42) fibril structure illuminates self-recognition and replication of amyloid in Alzheimer's disease." Nat Struct Mol Biol **22**(6): 499-505.

Yang, Y., D. Arseni, W. J. Zhang, M. Huang, S. Lovestam, M. Schweighauser, A. Kotecha, A. G. Murzin, S. Y. Peak-Chew, J. Macdonald, I. Lavenir, H. J. Garringer, E. Gelpi, K. L. Newell, G. G. Kovacs, R. Vidal, B. Ghetti, B. Ryskeldi-Falcon, S. H. W. Scheres and M. Goedert (2022). "Cryo-EM structures of amyloid-β 42 filaments from human brains." Science **375**(6577): 167-172.

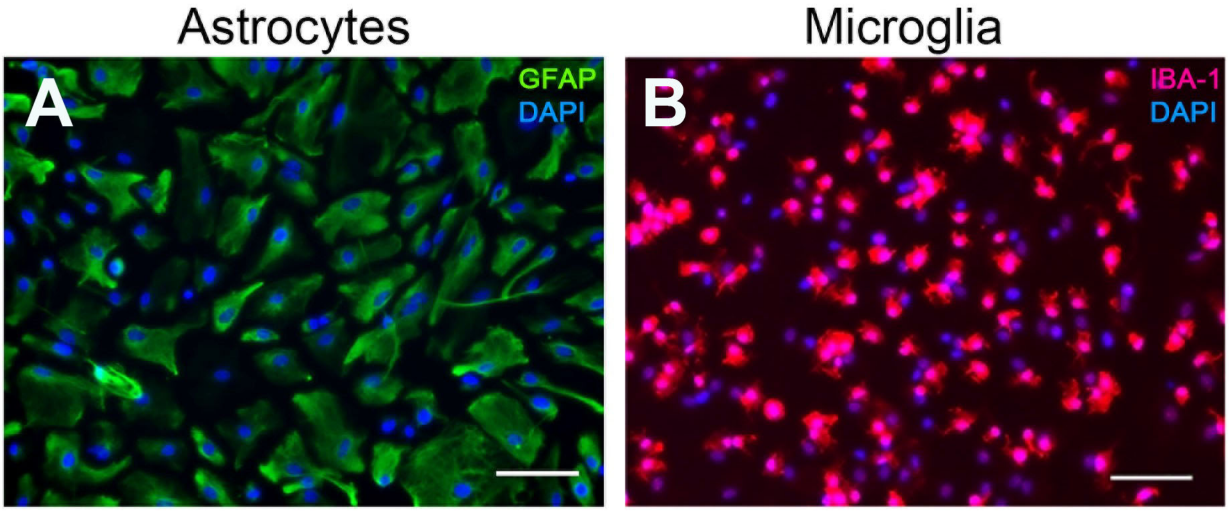

Supplemental Figure S2. Cell specific marker labeling of primary glial cultures. (**A**) Primary cultures of rat astrocytes were labeled with DAPI (blue) and an antibody to GFAP (green). (**B**) Primary cultures of rat microglia were labeled with DAPI (blue) and an antibody to Iba-1 (red). Cell counting revealed that cultures of each cell type were >99% positive for the corresponding marker.

Sample	CT1	CT2	CT3	Astrocyte Aβ42-1	Astrocyte Aβ42-2	Astrocyte Aβ42-3	Astrocyte Aβ40-1	Astrocyte Aβ40-2	Astrocyte Aβ40-3
CT1	1.0000	0.9544	0.7917	0.5711	0.6400	0.7504	0.7212	0.8165	0.8241
CT2	0.9544	1.0000	0.8558	0.5482	0.6130	0.7315	0.6765	0.7681	0.7927
CT3	0.7917	0.8558	1.0000	0.4436	0.5182	0.6663	0.5446	0.6425	0.7130
Astrocyte Aβ42-1	0.5711	0.5482	0.4436	1.0000	0.9865	0.9228	0.9634	0.9070	0.8545
Astrocyte Aβ42-2	0.6400	0.6130	0.5182	0.9865	1.0000	0.9634	0.9768	0.9470	0.9131
Astrocyte Aβ42-3	0.7504	0.7315	0.6663	0.9228	0.9634	1.0000	0.9568	0.9722	0.9763
Astrocyte Aβ40-1	0.7212	0.6765	0.5446	0.9634	0.9768	0.9568	1.0000	0.9729	0.9325
Astrocyte Aβ40-2	0.8165	0.7681	0.6425	0.9070	0.9470	0.9722	0.9729	1.0000	0.9759
Astrocyte Aβ40-3	0.8241	0.7927	0.7130	0.8545	0.9131	0.9763	0.9325	0.9759	1.0000

Sample	CT1	CT2	CT3	Microglia Aβ42-1	Microglia Aβ42-2	Microglia Aβ42-3	Microglia Aβ40-1	Microglia Aβ40-2	Microglia Aβ40-3
CT1	1.0000	0.9890	0.9907	0.6945	0.6781	0.6721	0.6476	0.6548	0.6605
CT2	0.9890	1.0000	0.9973	0.7261	0.7083	0.6975	0.6767	0.6890	0.6937
CT3	0.9907	0.9973	1.0000	0.7087	0.6930	0.6802	0.6595	0.6691	0.6746
Microglia Aβ42-1	0.6945	0.7261	0.7087	1.0000	0.9964	0.9545	0.9357	0.9521	0.9572
Microglia Aβ42-2	0.6781	0.7083	0.6930	0.9964	1.0000	0.9590	0.9405	0.9543	0.9584
Microglia Aβ42-3	0.6721	0.6975	0.6802	0.9545	0.9590	1.0000	0.9854	0.9863	0.9856
Microglia Aβ40-1	0.6476	0.6767	0.6595	0.9357	0.9405	0.9854	1.0000	0.9928	0.9934
Microglia Aβ40-2	0.6548	0.6890	0.6691	0.9521	0.9543	0.9863	0.9928	1.0000	0.9977
Microglia Aβ40-3	0.6605	0.6937	0.6746	0.9572	0.9584	0.9856	0.9934	0.9977	1.0000

Supplemental Table S 1. The *r* values of a Pearson correlation between control (untreated cultures) and fibril treated cultures for astrocytes and microglia. A value of 0.8< r <1, was considered as a very strong relationship, 0.6< r <0.79 was considered as a strong relationship, 0.4< r <0.59 was considered as a moderate relationship).

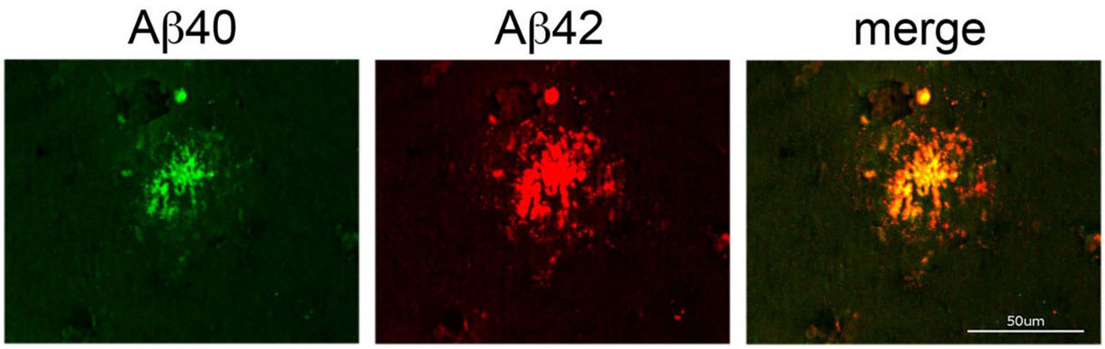

Supplemental Figure S3. Aβ species immunolabeling of parenchymal plaques in rTgF344-AD rat brain. Amyloid plaques in brain tissue sections from 15 months old rTgF344-AD rats were immunolabeled with an antibody to Aβ40 (green) and an antibody to Aβ42 (red). Scale bar = 50 μm. The amyloid plaques are mainly composed of fibrillar

Aβ42. Further, ELISA data from rTgF344-AD rat cortical tissue indicated that ≈70% of insoluble Aβ species was composed of Aβ42 (not shown).
